# A Novel Activatable Nanoradiosensitizer for Second Near‐Infrared Fluorescence Imaging‐Guided Safe‐Dose Synergetic Chemo‐Radiotherapy of Rheumatoid Arthritis

**DOI:** 10.1002/advs.202308905

**Published:** 2024-02-28

**Authors:** Yarong Jin, Dongsheng Li, Xiaochun Zheng, Mengting Gao, Wenxuan Wang, Xin Zhang, Weiwei Kang, Chongqing Zhang, Shutong Wu, Rong Dai, Ziliang Zheng, Ruiping Zhang

**Affiliations:** ^1^ Department of Radiology Fifth Hospital of Shanxi Medical University (Shanxi Provincial People's Hospital) Taiyuan 030000 China; ^2^ Research Team of Molecular Medicine First Hospital of Shanxi Medical University Shanxi Medical University Taiyuan 030001 China; ^3^ Department of Orthopedics Third Hospital of Shanxi Medical University (Shanxi Bethune Hospital) Taiyuan 030032 China; ^4^ Department of Radiology Shanxi Province Cancer Hospital (Shanxi Hospital Affiliated to Cancer Hospital Chinese Academy of Medical Sciences/Cancer Hospital Affiliated to Shanxi Medical University) Taiyuan 030013 China

**Keywords:** activatable nanoradiosensitizer, low‐dose radiotherapy, rheumatoid arthritis, second near‐infrared fluorescence imaging

## Abstract

The precise theranostics of rheumatoid arthritis (RA) remains a formidable challenge in clinical practice. Exploring novel applications of contemporary therapeutic approaches like chemo‐radiotherapy is promising as a highly effective strategy for RA. Herein, a novel activatable nanoradiosensitizer‐40 (denoted as IRnR‐40) is developed, based on encapsulating the clinically approved drugs cisplatin (DDP) and indocyanine green (ICG) within a gelatin shell to achieve second near‐infrared fluorescence (NIR‐II FL) imaging‐guided safe‐dose synergetic chemo‐radiotherapy. The high concentration of matrix metalloproteinase‐9 (MMP‐9) in the RA microenvironment plays a pivotal role in triggering the responsive degradation of IRnR‐40, leading to the rapid release of functional molecules DDP and ICG. The released ICG serves the dual purpose of illuminating the inflamed joints to facilitate accurate target volume delineation for guiding radiotherapy, as well as acting as a real‐time reporter for quantifying the release of DDP to monitor efficacy. Meanwhile, the released DDP achieves highly effective synergistic chemotherapy and radiosensitization for RA via the dual reactive oxygen species (ROS)‐mediated mitochondrial apoptotic pathway. To sum up, this activatable nanoradiosensitizer IRnR‐40 is believed to be the first attempt to achieve efficient NIR‐II FL imaging‐guided safe‐dose chemo‐radiotherapy for RA, which provides a new paradigm for precise theranostics of refractory benign diseases.

## Graphical abstract

In this work, a novel activatable nanoradiosensitizer (IRnR‐40) is developed, based on encapsulating the clinically approved drugs of cisplatin and indocyanine green in gelatin to achieve precisely responsive second near‐infrared fluorescence imaging‐guided safe chemo‐radiosynovectomy for the advanced stage of RA, which provides a new paradigm for precise theranostics of refractory benign diseases.

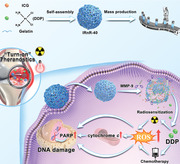

## 1 Introduction

Rheumatoid arthritis (RA), called “undead cancer”, is an autoimmune inflammatory disease related to persistent synovitis. Abnormal proliferation of rheumatoid arthritis synovial fibroblasts (RASF) is recognized as one of the key pathological characteristics of synovitis and secretes large amounts of pro‐inflammatory factors and matrix metalloproteinases (MMPs).^[^ ^1^, ^2^ ^]^ Gelatinase MMP‐9, as one of the most important MMPs, plays a critical role in RA inflammation, articular destruction, and bone erosion. It is abnormally highly expressed in RA synovial tissue and synovial fluid, and positively correlated with disease progression and severity of RA.^[^ ^3^, ^4^ ^]^ The primary approach of RA involves the use of chemical drugs, such as nonsteroidal anti‐inflammatory drugs (NSAIDs), disease‐modifying anti‐RA drugs (DMARDs), and glucocorticoids (GCs), to alleviate symptoms and control the progression of arthritis. However, due to the nonspecific targeting and poor bioavailability, most of these drugs produce various adverse effects and have limited efficacy, especially for the advanced stage of RA.^[^ ^5^ ^]^ It is thus urgently desirable to develop a novel theranostic strategy, which aims at inducing the damage of proliferative synovial fibroblasts for severe RA.

Radiation therapy (RT) has long been the cornerstone of malignant tumor therapy, but it is also utilized for benign pathologies.^[^ ^6^, ^7^ ^]^ Radiosynovectomy is a well‐established therapy for several acute and chronic inflammatory joint disorders and involves injecting beta rays‐emitting radionuclide intra‐articularly for excessive reactive oxygen species (ROS) production to destroy the proliferative synovium.^[^ ^8^ ^]^ Unfortunately, complex techniques and advanced equipment to operate restrict its popularity in clinical applications. Notably, low‐dose X‐ray radiotherapy (LD‐RT) has been used clinically for autoimmune illnesses.^[^ ^9^ ^]^ Studies have shown that LD‐RT has excellent anti‐inflammatory and immune modulation properties to ameliorate the pain and reduce bone destruction of RA.^[^ ^10^, ^11^, ^12^ ^]^ However, the limited efficacy and suboptimal clinical application of traditional LD‐RT can be attributed to radioresistance, inevitable injuries to normal tissues, and the worry about producing radiation‐induced tumors.^[^ ^13^ ^]^ Therefore, the precise delineation of hyperplastic synovium and the development of efficient radiosensitizers have been reckoned as two critical factors for exploring a novel, safe synovectomy to enhance the efficacy of radiotherapy for RA.

Traditional image‐guided radiotherapy (IGRT) with cone‐beam computed tomography (CBCT) has certain inherent limitations, mostly revolving around inadequate image resolution and difficult real‐time dynamic monitoring of disease.^[^ ^14^ ^]^ Inspiringly, the emerging second near‐infrared fluorescence (NIR‐II FL, 1000–1700 nm) imaging in vivo is characterized by real‐time, superior spatial resolution, and dynamic tracking of the changes in interest regions, which holds the potential to precisely delineate the target volume to guide radiotherapy.^[^ ^15^, ^16^ ^]^ Currently, the biocompatible indocyanine green (ICG) has gained widespread employment in NIR‐II FL imaging in clinical practice due to its tailing emission in the NIR‐II window.^[^ ^17^, ^18^ ^]^ Interestingly, nano‐sized ICG can be passively targeted for NIR‐II FL imaging to enable real‐time dynamic monitoring of RA. Nonetheless, distinguishing lesion sites from surrounding normal tissues using ICG via NIR‐II FL imaging proves challenging, necessitating heightened specificity and sensitivity. The “activatable” strategy provides significant benefits for the development of imaging nanoprobes, as it facilitates responsive “turn on” NIR‐II FL in the unique microenvironment of the lesion site.^[^ ^19^ ^]^ Therefore, endowing ICG with an activatable feature could offer a promising approach to enhance the specificity and sensitivity in localizing the abnormally proliferative synovial tissues of RA, making it a preferential choice for precise target delineation in radiotherapy guidance.

Recently, the extraordinary advantages of multifunctional nanoprobes have been mainly reflected in theranostic clinical application.^[^ ^20^ ^]^ The vigorous development of nanotechnology has provided a new direction for exploring safe and effective radiosensitizing nanomaterials. Encouragingly, a variety of metal‐based (high atomic number (Z) elements, including Pt, Hf, and Au) nanoradiosensitizers with efficient delivery performance have stepped into the clinical trial, even phase 2–3 stages, indicating the great application potential of nanoradiosensitizers in the clinic.^[^ ^21^, ^22^, ^23^ ^]^ Cisplatin (DDP) containing the high‐Z element Pt, significantly intensifies DNA damage induced by ROS and has been widely used in tumor radiosensitization therapy.^[^ ^24^ ^]^ Additionally, DDP, as one of the most frequently employed chemotherapy drugs, can produce interstrand and intrastrand cross‐linking with DNA and interfere with DNA repair, inducing DNA damage and apoptosis of cancer cells.^[^ ^25^ ^]^ In combination with radiotherapy, DDP could enhance the ROS‐amplified cell death in the target volume. However, like most chemotherapy drugs, the clinical application of DDP is restricted to many dose‐limiting side effects, including renal toxicity, neurotoxicity, and bone marrow suppression.^[^ ^25^ ^]^ To solve the problem, activatable nanomedicines are employed to reduce the systemic toxicity of traditionally intravenous drugs, thus significantly increasing therapeutic benefits against refractory diseases.^[^ ^26^, ^27^ ^]^ For example, Xiao and colleagues developed a self‐targeting platinum(IV)‐lactose prodrug‐based nano‐assembly (Pt STNA) that responsively delivered cisplatin(II) into cancer cells to achieve safe and efficient chemo‐radiotherapy for hepatocellular carcinoma.^[^ ^28^ ^]^ Despite significant progress, the development of activatable theranostic nanoradiosensitizers with excellent potential for clinical translation to achieve precise target imaging and image‐guided synergistic chemo‐radiotherapy remains a formidable challenge.

**Scheme 1:**
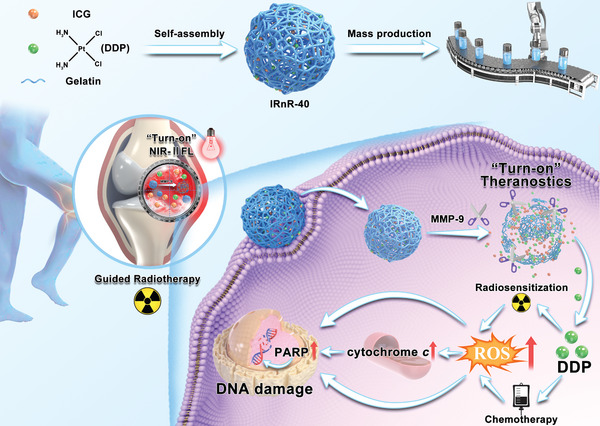
Schematic illustration of the IRnR‐40 preparation, activatable drug release, and NIR‐II FL imaging‐guided safe‐dose chemo‐radiotherapy.

In this study, we proposed an innovative chemo‐radiotherapy strategy for the development of versatile nanoradiosensitizers using clinically approved drugs and endogenous substances. This nanoradiosensitizer was designed to selectively activate its theranostics functions to clear the proliferative synovial tissues while sparing adjacent healthy tissues, thus enabling safe‐dose precise synovectomy in the advanced stage of RA. To this end, we developed a novel enzyme‐responsive nanoradiosensitizer‐40 (denoted as IRnR‐40), which ingeniously encapsulated DDP and ICG in a gelatin (gel) shell for achieving precisely activatable NIR‐II FL imaging‐guided safe chemo‐radiosynovectomy (**Scheme** **1**). It is worth mentioning that the gel, a biological endogenous substance, is Food and Drug Administration (FDA)‐approved for application in medical, pharmaceutical, and food industries.^[^ ^29^, ^30^ ^]^ Within the inflammatory microenvironment, the IRnR‐40 could be passively targeted to the inflamed joints through the extravasation through leaky vasculature and subsequent inflammatory cell‐mediated sequestration (ELVIS) effect.^[^ ^31^ ^]^ Meanwhile, the high concentration of MMP‐9 facilitated the responsive degradation of the gel shell, leading to the efficient release of functional small molecules of DDP and ICG from IRnR‐40.^[^ ^32^ ^]^ The released ICG rapidly restored NIR‐II FL signaling and served as a reporter for the quantity of released DDP and the target volume ready for radiotherapy. Simultaneously, DDP, a well‐established chemotherapeutic agent, was utilized to explore its potential for radiosensitization and chemotherapy by destroying proliferative synovial fibroblasts in RA. The high levels of ROS in RA could trigger the mitochondrial caspases‐cascade pathway to promote RASF apoptosis, which ultimately restrained synovial hyperplasia and cartilage destruction. To sum up, our study represents the pioneering effort to demonstrate the feasibility of DDP with a “teaching old drugs new tricks” strategy for RA treatment, and the practicality of activatable NIR‐II FL imaging‐guided safe‐dose chemo‐radiotherapy, which may pave the way for exploring highly efficient RA theranostic strategies.

## 2 Results and Discussion

**Figure 1:**
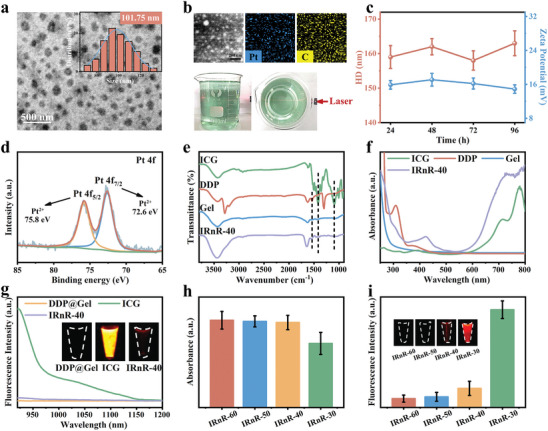
Fabrication and characterization of IRnR‐40. a) Representative TEM image of IRnR‐40. Inset: TEM‐measured size distribution of IRnR‐40. b) The top photos: the corresponding elemental mapping (Pt and C) of IRnR‐40. The bottom photos: the mass‐produced nanomedicines and the Tyndall effect observed by passing a laser beam through the IRnR‐40 aqueous solution. c) The hydrodynamic diameter (HD) and zeta potential of IRnR‐40 at different time points. d) XPS spectrum of Pt in the IRnR‐40. e) FTIR spectra of ICG, DDP, Gel, and IRnR‐40. f) UV–vis absorption of ICG, DDP, Gel, and IRnR‐40. g) Excitation spectra of DDP@Gel, free ICG, and IRnR‐40 with equal ICG concentration (50 µg mL^−1^). Insets: fluorescence images of DDP@Gel, free ICG, and IRnR‐40 under 808 nm excitation. h) UV–vis absorption at 309 nm and i) NIR‐II fluorescence intensity of IRnR with different DDP/ICG mass ratios (IRnR‐60: DDP/ICG = 60; IRnR‐50: DDP/ICG = 50; IRnR‐40: DDP/ICG = 40; IRnR‐30: DDP/ICG = 30). Mean ± SD (n = 3).

The IRnR‐40 was successfully fabricated via a simple one‐step strategy, utilizing the inherent property in the self‐assembly processes of gels to encapsulate both ICG and DDP.^[^ ^33^ ^]^ The as‐synthesized IRnR‐40 was initially analyzed by transmission electron microscopy (TEM) imaging (**Figure** **1**a), revealing a spherical and uniformly sized structure with an average diameter of 101.75 nm. Correspondingly, elemental mapping analyses were conducted to further verify the presence of Pt in the IRnR‐40 (Figure 1b, top photos). Remarkably, a bulk solution of IRnR‐40 exhibited a strong Tyndall effect and remained well‐dispersed without any noticeable sedimentation after a long time placement even for one week, indicating that the IRnR‐40 had the potential for mass‐produced clinical applications (Figure 1b, bottom photos). The IRnR‐40 could be dispersed well in deionized water, PBS, Dulbecco's modified Eagle's medium (DMEM), and fetal bovine serum (FBS) after 24 h with good water‐solubility and transmission. No obvious changes in the hydrodynamic diameter (HD) and zeta potential of IRnR‐40 were observed in different solutions (Figure S1, Supporting Information). Meanwhile, as depicted in Figure 1c, IRnR‐40 exhibited no significant changes in HD and zeta potential (160.5 nm and +16.0 mV) over 96 h, suggesting that the IRnR‐40 had excellent stability in physiological media. To determine the elemental composition and chemical valence of IRnR‐40, X‐ray photoelectron spectroscopy (XPS) analysis was carried out. As depicted in Figure 1d, the XPS spectrum confirmed the existence of the Pt element as a representative of DDP in the IRnR‐40, which was consistent with the results of elemental mapping analysis. The Fourier‐transform infrared (FTIR) spectra were conducted to validate the effective encapsulation of DDP and ICG within the gel. As presented in Figure 1e, the spectrum of IRnR‐40 revealed characteristic peaks related to amine stretching (3400–3200 cm^−1^), symmetric amine bending (1300–1200 cm^−1^), and asymmetric amine bending (1600–1500 cm^−1^) of DDP.^[^ ^34^ ^]^ The ICG corresponded to C─N stretching at 1104 cm^−1^ and CH_3_ asymmetric bending at 1424 cm^−1^. More importantly, the characteristic peak of IRnR‐40 revealed a decrease in the COO─ stretching vibration of the gel at 1148 cm^−1^ after the incorporation of DDP.^[^ ^35^ ^]^ This observation suggests a chemical coupling between some of the COOH groups in the gel and the Pt atoms of DDP, resulting in a reduction of free COOH groups. Furthermore, the UV–vis absorbance spectrum of IRnR‐40 displayed distinct absorption peaks for DDP and ICG at 309 and 780 nm, respectively, thus providing strong evidence for the successful construction of the IRnR‐40 (Figure 1f).

Based on the NIR‐II FL imaging performance of ICG, we then studied the fluorescence property of IRnR‐40. Compared to free ICG, the NIR‐II FL of IRnR‐40 was effectively “off” due to the artful entrapment of ICG molecules within the gel matrix, inducing aggregation‐caused fluorescence quenching (Figure 1g). This approach holds promise for achieving activatable NIR‐II FL imaging upon release of free ICG from IRnR‐40. In order to simultaneously build maximal loading efficiency of DDP and optimal fluorescence quenching, a series of nanomedicines IRnR‐x with different DDP/ICG mass ratios (x) were synthesized to investigate their UV–vis absorption at 309 nm and NIR‐II FL emission properties (Figure 1h,i). Among these formulations, IRnR‐40, characterized by a DDP/ICG ratio of 40:1, exhibited the highest DDP loading capacity and the best fluorescence quenching effect. Consequently, the IRnR‐40 was selected as the ideal candidate for the following experiments.

**Figure 2:**
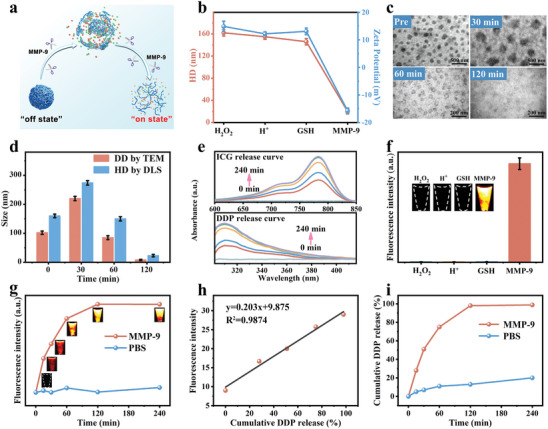
Evaluation of ICG and DDP release from IRnR‐40 in vitro. a) Schematic degradation process of IRnR‐40. b) The HD and zeta potential changes of IRnR‐40 under different conditions. c) Morphological changes and d) size changes of IRnR‐40 after incubation with MMP‐9 for 0, 30, 60, and 120 min at 37 °C. DD: dehydrated diameter. e) UV–vis absorption of released ICG and DDP from IRnR‐40 after incubation with MMP‐9 for 0, 15, 30, 60, 120, and 240 min at 37 °C. f) NIR‐II FL response property of IRnR‐40 toward various stimuli. g) NIR‐II FL intensity and images of IRnR‐40 in the presence and absence of MMP‐9 at different time points. h) The linear relationship between the cumulatively released DDP and the fluorescence intensity of IRnR‐40 in the presence of MMP‐9. i) Time‐dependent profiles of the cumulative release of Pt in the presence and absence of MMP‐9 by ICP‐OES analysis. Mean ± SD (n = 3).

The IRnR‐40 experienced rapid degradation of the gel shell upon exposure to elevated MMP‐9 concentration in the RA microenvironment, resulting in the controllable release of functional small molecules (**Figure** **2**a). First, to verify the specific response of IRnR‐40 to various stimuli, including H_2_O_2_, H^+^, GSH, and MMP‐9, each was individually introduced to the IRnR‐40. As expected, in the presence of MMP‐9, the HD of IRnR‐40 significantly decreased from 162.0±5.7 to 20.0±4.5 nm, and the zeta potential displayed a remarkable charge reversal, jumping from +14.9 to −15.7 mV. However, the size and zeta potential of IRnR‐40 remained almost unchanged in the case of H_2_O_2_, H^+^, and GSH (Figure 2b), indicating its selective responsiveness to MMP‐9‐induced structural decomposition. The morphological alterations of IRnR‐40 with the addition of MMP‐9 were demonstrated by TEM and dynamic light scattering (DLS) analyses. Initially, the structure of IRnR‐40 exhibited rapid swelling behavior, followed by a gradual reduction in size, and near‐complete degradation within 120 min (Figure 2c,d). This process facilitated the drug penetration into the core region at arthritic sites. Moreover, we investigated the effect of MMP‐9 in a wide concentration distribution on the responsive size changes of IRnR‐40 (Figure S3, Supporting Information). The findings revealed a gradual decrease in the size of IRnR‐40 in response to increasing MMP‐9 concentration, indicating the high sensitivity of IRnR‐40 for MMP‐9. With both ICG and DDP co‐loaded into IRnR‐40, the release curves showed that almost 50% of the accumulated ICG and DDP were released in the first 30 min, reaching the maximum release levels at 120 min (Figure 2e). To investigate the specific “off‐on” switchable NIR‐II FL properties, the selectivity of IRnR‐40 in response to different simulated conditions of the RA microenvironment was recorded (Figure 2f). Notably, the introduction of MMP‐9 into the IRnR‐40 solution promptly triggered a specific “turn‐on” process of NIR‐II FL. Furthermore, the time‐dependent fluorescence responses of IRnR‐40 in the presence of MMP‐9 exhibited a similar tendency to the ICG release curve in Figure 2g. All of the above phenomena indicate that the IRnR‐40 could achieve highly selective MMP‐9 response degradation.

We further conducted a correlation study to examine the relationship between the NIR‐II FL intensity of IRnR‐40 in response to MMP‐9 and the cumulative released amount of DDP (Figure 2h). The result revealed a well‐fitted linear equation: y = 0.203x+9.875 (R^2^ = 0.9874), showing that the change in NIR‐II FL signal may serve as a precisely quantitative indicator of DDP release from IRnR‐40. After calculating the concentration standard curve, it was determined that the loading efficiency of DDP within IRnR‐40 was 20.74%. Moreover, the encapsulation efficiency was found to be 90.61 ± 1.91% when 5 mg mL^−1^ of DDP was added to the solution (Figure S5, Supporting Information). We then delved into this by investigating the release of Pt ions at various time points via inductively coupled plasma‐optical emission spectrometry (ICP‐OES) analysis (Figure 2i). The free Pt ions cumulatively released from the IRnR‐40 with MMP‐9 addition displayed a noticeable upward trend within 120 min, confirming that the DDP was continuously and efficiently generated from the IRnR‐40 degradation triggered via the MMP‐9. As is well known, a routine dose of DDP is associated with serious side effects that hinder its clinical application. This innovative approach of IRnR‐40 (DDP‐coated gel) holds significant promise to minimize the toxicity of DDP to healthy tissues and maximize its therapeutic efficacy in vivo at a safe dose. In addition, the responsive release of ICG from IRnR‐40 resulted in the rapid recovery of NIR‐II FL, thus enabling the real‐time quantitative monitoring of DDP release.

**Figure 3:**
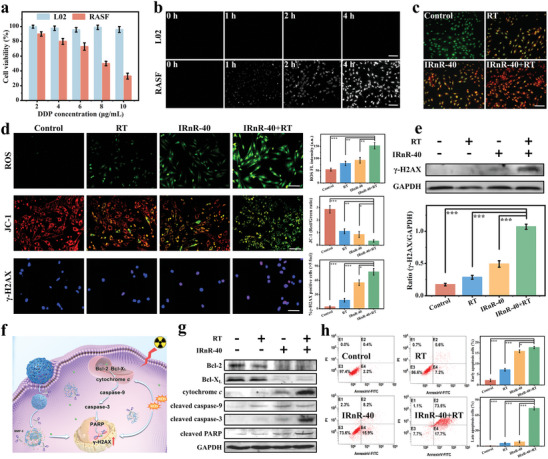
Therapeutic efficacy of IRnR‐40 in vitro. a) Cell viability of L02 and RASF cells treated with IRnR‐40 at different concentrations for 24 h. b) Microscopic NIR‐II FL images of L02 and RASF cells treated with IRnR‐40 for 0, 1, 2, and 4 h. Scale bar: 100 µm. c) The morphological study of cell apoptosis using AO/EB staining. Scale bar: 100 µm. d) The fluorescence images of intracellular total ROS obtained by DCFH‐DA staining, mitochondrial membrane potential (MMP) by JC‐1 staining, and DSBs by γ‐H2AX immunofluorescence following various treatments. Scale bar: 100 µm. Quantitation of ROS, JC‐1 aggregate (red)/JC‐1 monomer (green) fluorescence intensity, and the percentage of cells with >5 γ‐H2AX‐labeled foci. e) The level of γ‐H2AX protein expression in RASF cells assessed by western blot assay. The gray density scanning analysis of γ‐H2AX protein. f) Schematic illustration of RASF cells treated with IRnR‐40 plus RT and the related apoptosis pathway. g) Western blot analysis of the proteins involved in apoptosis in RASF cells. h) Flow cytometry detection of apoptotic cells and the proportion of early and late apoptosis cells. Mean ± SD (n = 3). ^*^ *p* < 0.05, ^**^ *p* < 0.01, ^***^ *p* < 0.001. Student's t‐test.

Encouraged by the promising physicochemical properties of IRnR‐40, we first conducted a Cell Counting Kit‐8 (CCK‐8) assay to evaluate the cytotoxicity of IRnR‐40 on human normal liver cells (L02) and rheumatoid arthritis synovial fibroblasts (RASF) at various concentrations. As seen in **Figure** **3**a, concentration‐dependent cytotoxicity of IRnR‐40 was observed in RASF cells. However, there was no apparent cytotoxicity in L02 cells at all tested concentrations. This is attributed to the significantly higher concentration and enhanced enzyme activity of MMP‐9 in RASF cells compared to that in L02, as demonstrated by gel zymography (Figure S6, Supporting Information), thus triggering the specific release of DDP from the IRnR‐40 degradation in RASF cells. The increasing NIR‐II FL signal further indicated the specific cellular uptake of IRnR‐40 in RASF cells rather than in L02 with maximum fluorescence intensity at 4 h (Figure 3b). This is suggestive of the maximum accumulation of DDP according to the linear relationship between the recovered NIR‐II FL intensity of IRnR‐40 and the released DDP amount. In addition, we assessed the cytotoxicity of RT on RASF cells after exposure to X‐ray irradiation ranging from 0 to 10 Gy (Figure S7, Supporting Information). The cell viability remained above 80% for X‐ray doses below 1 Gy, while cells at 2–10 Gy elicited significant cytotoxicity with a dose‐dependent property. Remarkably, the IRnR‐40 exhibited an excellent radiosensitization effect on cells at 0.5–1 Gy dose irradiation (Figure S8, Supporting Information). Furthermore, the living/dead cell assays of RASF cells were conducted by the dual acridine orange‐ethidium bromide (AO/EB) staining (Figure 3c). The IRnR‐40+RT group showed the highest level of cell apoptosis when compared to the other groups, which was consistent with the results of the CCK‐8 tests.

It is well known that ROS is closely associated with cellular oxidative stress and excessive ROS can lead to DNA and protein damage, ultimately leading to cell apoptosis or necrosis.^[^ ^36^ ^]^ Due to the transient and reactive nature of ROS, ROS‐enhancing treatments such as photodynamic and sonodynamic therapy could achieve timely hyperplastic synovium excision in a site‐localized manner to improve the efficacy of RA.^[^ ^37^, ^38^, ^39^ ^]^ The generation of ROS upon ionizing radiation is a critical driver in cell death.^[^ ^40^ ^]^ Owing to the radiosensitive property of IRnR‐40, we proceeded to investigate the ROS levels using a 2′,7′‐dichlorodihydrofluorescein diacetate (DCFH‐DA) assay. In Figure 3d, the intensity of the fluorescence signal in RASF cells treated with the IRnR‐40 plus RT was higher than that of X‐ray irradiation or IRnR‐40 alone, showing that the DDP‐mediated chemotherapy and radiosensitization synergistically enhanced ROS generation to eliminate pathological synovial cells. To further understand the impact on cellular mitochondria, we employed JC‐1 staining to analyze changes in mitochondrial membrane potential (MMP). Upon X‐ray irradiation, the treatment of RASF cells with IRnR‐40 resulted in a significant reduction in red fluorescence, indicating the disruption of the mitochondrial membrane caused by the excessive ROS levels.

DNA double‐strand breaks (DSBs) are well‐known as catastrophic events to cause severe damage to cells.^[^ ^41^ ^]^ We then tested the foci of DSBs through immunofluorescence staining and γ‐H2AX protein expression through western blot (Figure 3d,e). Significantly, a notable increase in both the number of foci and the γ‐H2AX protein levels was observed in the IRnR‐40+RT group, indicating the induction of more severe DSBs compared to the monotherapy group, ultimately leading to cellular apoptosis. Hence, the synergistic effect of safe‐dose chemo‐radiotherapy with dual ROS‐enhancing performance could remarkably kill RASF. This can be attributed to the increased X‐ray absorption by high‐Z atom Pt in IRnR‐40, leading to the conversion of H_2_O/O_2_ into ROS, thereby inducing DNA damage and mitochondrial destruction in the RASF cells (Figure 3f).

To comprehensively understand the mechanisms underlying RASF cell apoptosis, we performed an analysis of apoptosis‐associated proteins. As shown in Figure 3g, the combined therapy group (IRnR‐40+RT) demonstrated a significant increase in cytochrome *c* expression and a significant decrease in anti‐apoptotic proteins (Bcl‐2 and Bcl‐X_L_) expression in comparison to the groups treated with either IRnR‐40 or RT alone. This phenomenon was closely associated with mitochondrial fragmentation, culminating in the release of cytochrome *c* into the cytoplasm. Furthermore, we detected the cleavage and activation of key proteins, namely caspase‐9, caspase‐3, and PARP. Typically, caspase‐9 as the major initiator is usually the first to be activated in the mitochondrial apoptotic pathway and then converges to activate the crucial executioner caspase‐3, which subsequently cleaves its target protein PARP, finally leading to cell apoptosis.^[^ ^42^ ^]^ Accordingly, flow cytometric analysis yielded consistent results, clearly demonstrating significantly elevated percentages of both early and late apoptotic cells in the IRnR‐40+RT group compared to the other groups (Figure 3h). These results suggest unequivocally that excessive ROS leads to enhanced DSBs and mitochondrial damage, ultimately resulting in heightened cell apoptosis. Taken together, the above findings offer compelling evidence that the IRnR‐40 could be utilized as an activatable NIR‐II FL nanoprobe to efficiently excise hyperplastic synovium via the dual ROS‐mediated mitochondrial apoptotic pathway.

**Figure 4:**
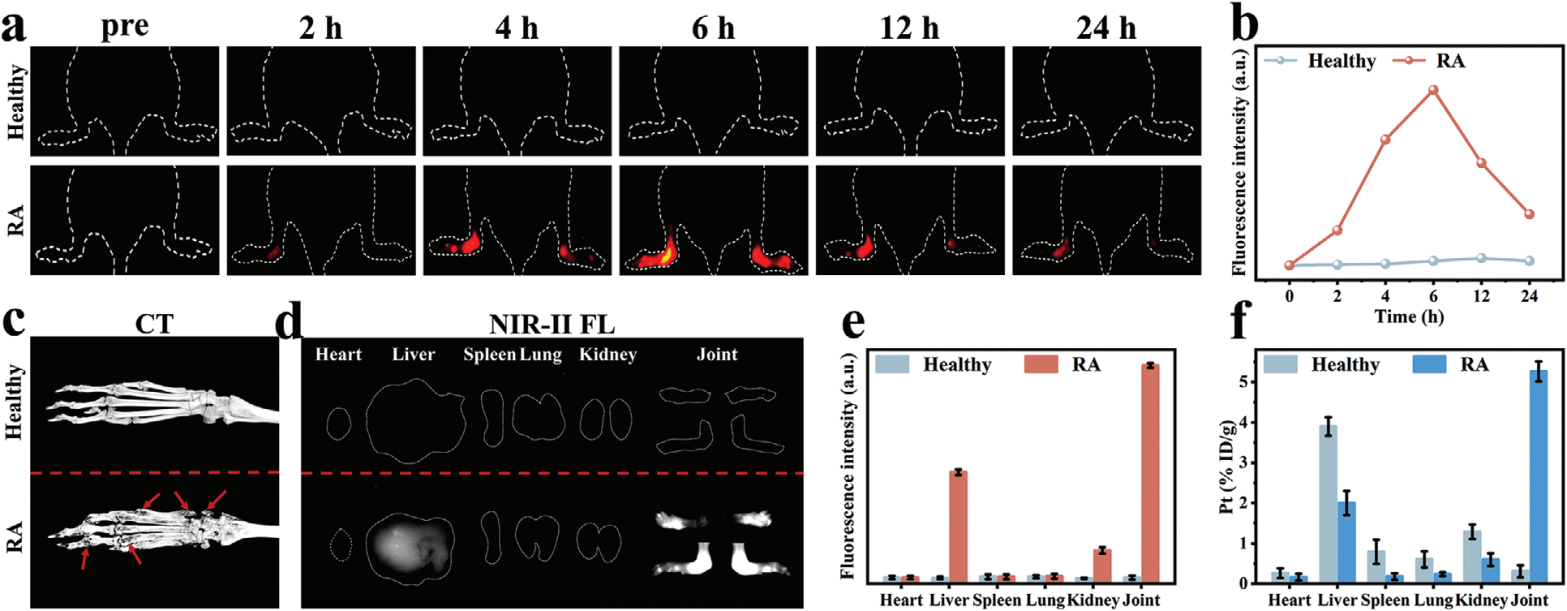
In vivo NIR‐II fluorescence imaging. a) Time‐dependent NIR‐II FL imaging of healthy and RA mice following intravenous injection of IRnR‐40 under 808 nm excitation, respectively. b) Time‐dependent fluorescence intensity changes were determined by the NIR‐II FL imaging of healthy and RA mice. c) Representative micro‐CT images of joints in healthy and RA groups. The red arrow refers to bone destruction. d) *Ex vivo* NIR‐II FL imaging, e) quantized values, and f) biodistribution of Pt in main organs and joints at 6 h post‐injection. Mean ± SD (n = 3).

As shown in **Figure** **4**a, the IRnR‐40 was able to specifically illuminate the inflamed joints in the RA group, indicating its superiority in accurately delineating the target volume from the adjacent normal tissues to guide radiotherapy. Quantitative analysis revealed that the intensity of NIR‐II FL signals in the inflamed joints gradually increased over time and peaked at 6 h after intravenous injection (Figure 4b), implying that this was the optimum time point for RT with the greatest accumulation of IRnR‐40. Conversely, no obvious “turn‐on” process of NIR‐II FL was observed in healthy mice. This is attributed to the passive “ELVIS” effect of IRnR‐40 and the substantially higher concentration of MMP‐9 to specifically trigger the IRnR‐40 degradation in the RA joints compared to the healthy counterparts. Considering the linear correlation between the NIR‐II FL intensity of ICG and the released amount of DDP (Figure 2h), we derived a kinetic curve of DDP released from IRnR‐40 injected intravenously (Figure S9, Supporting Information), indicating that ≈51.3% of DDP was released in RA microenvironment within the first 3 h, reaching its maximum release at 6 h in vivo. In addition, the reconstructed micro‐CT images of the inflamed joints exhibited severe bone erosion in contrast to the healthy group (Figure 4c), corroborating the observation through the NIR‐II FL imaging. *Ex vivo* fluorescence imaging and quantitative analysis further revealed that the IRnR‐40 accumulated mainly in inflamed joints, followed by the liver, reflecting a predominant hepatic metabolic pathway (Figure 4d,e). Moreover, we performed ICP‐OES analysis to quantitatively measure the Pt contents in the joints and major organs (Figure 4f). Consistent with the biodistribution of IRnR‐40, the RA group showed significantly increased levels of Pt elements within the inflamed joints compared to the healthy group. These findings indicated that the accumulation of IRnR‐40 in the inflamed joints could be actively degraded by highly expressed MMP‐9 to release DDP and ICG, thereby facilitating NIR‐II FL imaging‐guided safe‐dose chemo‐radiotherapy in RA.

**Figure 5:**
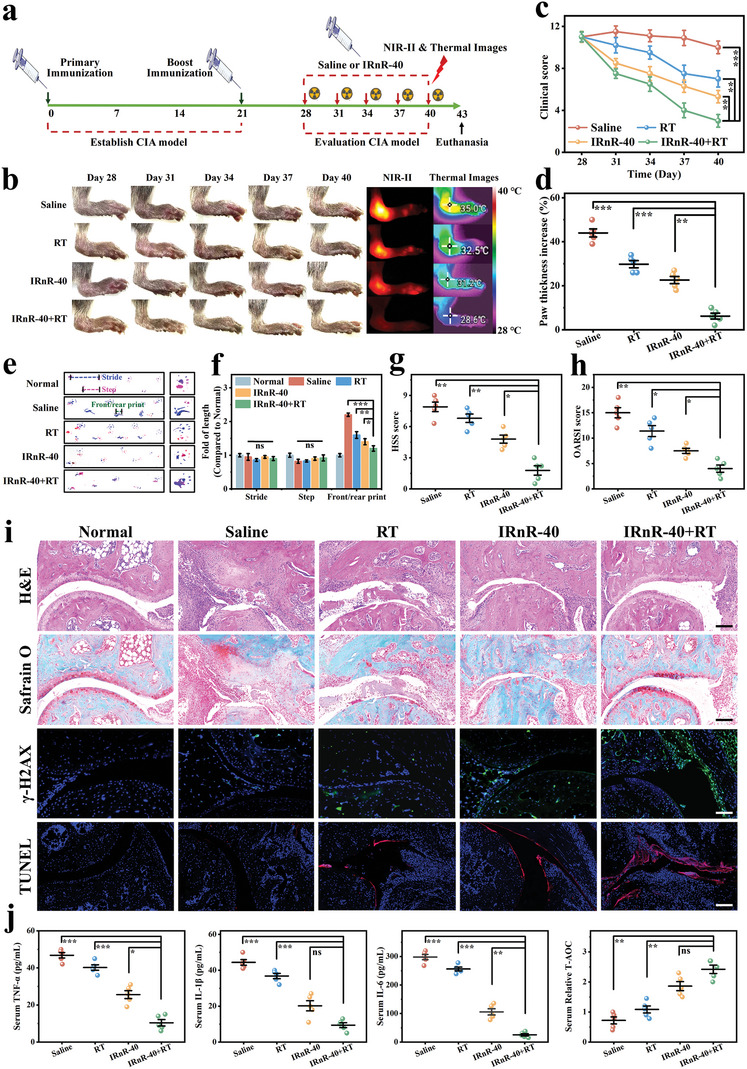
Therapeutic efficacy of safe‐dose chemo‐radiotherapy in vivo. a) Outline of the experimental procedure. b) Representative images, NIR‐II FL images, and thermal images of arthritic paws in different groups. c) Clinical score and d) paw thickness changes in different groups. e) Gait analysis for various treatments, with the blue dotted line representing stride length, the red dotted line representing step length, and the green dotted line representing the length of front/rear paw prints. Red prints, fore paws; blue prints, hind paws. f) Quantification of stride length, step length, and the length of front/rear paw prints at the end of the treatments. g) The HSS scores and h) the modified OARSI scores from different treatment groups. i) Histological analysis using H&E, Safranin‐O, γ‐H2AX immunofluorescence, and TUNEL staining of joint slices in different groups. Scale bar: 100 µm. j) Serum concentrations of cytokines (TNF‐α, IL‐1β, and IL‐6) and total antioxidant capacity (T‐AOC) test of serum. Mean ± SD (n = 5). ^*^ *p*<0.05, ^**^ *p*<0.01, ^***^ *p*<0.001, ns = not significant. Student's t‐test.

Inspired by the exciting in vitro results above, we further assessed the synergistically therapeutic efficacy of the safe‐dose chemo‐radiotherapy in the advanced stage (arthritic severity score>8)of the collagen‐induced arthritis (CIA) model following the treatment scheme outlined in **Figure** **5**a. A total of four groups including 1) Saline, 2) RT, 3) IRnR‐40, and 4) IRnR‐40+RT were randomly set up from day 28 since the first immunization. As shown in Figure 5b,c, the control group treated with saline exhibited severe paw erythema and swelling, with the arthritis scores consistently remaining high throughout the treatment period. These symptoms were partially alleviated in the IRnR‐40 or RT group alone. Of note, the most efficient anti‐inflammatory effect and the lowest clinical score were observed in the IRnR‐40+RT group. Moreover, the results of NIR‐II FL imaging and corresponding quantitative analyses at the end of the different treatments indicated that the fluorescence intensity in the inflamed joints corresponded to the severity of RA (Figure 5b; Figure S10, Supporting Information). The swelling and hyperthermia of paws, as recorded by thermography, were almost completely relieved in mice treated with the IRnR‐40+RT, indicating that DDP‐mediated radiosensitization and chemotherapy endowed IRnR‐40 with the strongest anti‐arthritis performance in vivo. The measurement of paw thickness as an indicator of arthritis progression yielded consistent results (Figure 5d). The micro‐CT was then employed to assess the bone damage of the inflamed joints in different groups. Saline‐treated mice showed the most severe bone erosion, whereas the combined treatment almost alleviated the injury of advanced‐stage arthritis. Furthermore, quantitative analysis of the interest region showed that IRnR‐40+RT successfully maintained bone mass in terms of ratio of bone volume to tissue volume (BV/TV), bone mineral density (BMD), ratio of bone surface area to bone volume (BS/BV), trabecular number (Tb.N), trabecular thickness (Tb.Th), and trabecular separation (Tb.Sp) (Figure S11, Supporting Information), performing significantly better than the monotherapy group. The athletic functionality of mice was assessed by allowing each mouse to move freely from one side to the other, marking the fore paws with red ink and the hind paws with blue ink to record their footprints. In the normal groups, the prints of the front and hind paws were overlapping, whereas those in the saline group exhibited distinct patterns. Remarkably, the mice treated with the combined treatment almost fully recovered their movement ability (Figure 5e,f). The above results suggest that synergistic safe‐dose chemo‐radiotherapy is an optimal mitigation strategy for the advanced stage of RA.

The model mice treated with saline showed substantial pannus formation and severe bone deterioration compared with the normal group, resulting in the highest histological synovitis scoring system (HSS) score^[^ ^43^ ^]^ (7.98 ± 0.46) on the examination of histology slides of ankle joints stained with hematoxylin and eosin (H&E). The RA mice treated with RT or IRnR‐40 alone had fairly lower levels of bone erosion, pannus formation, and synovial hyperplasia. Notably, the synergistic treatment group (IRnR‐40+RT) exhibited the least pathologic features and the lowest HSS score (2.84 ± 0.34) (Figure 5g,i). The findings were corroborated by Safranin‐O staining, which revealed glycosaminoglycans in cartilage. In the saline group, there was a significant loss of proteoglycan, indicating severe damage to the articular cartilage. Remarkably, the cartilage of mice subjected to combined treatment appeared almost intact, with the lowest Osteoarthritis Research Society International (OARSI) histological score^[^ ^44^ ^]^ (4.03 ± 0.29) (Figure 5h,i). In addition, the expression of γ‐H2AX protein in tissues was assessed through immunofluorescence. The results demonstrated that the IRnR‐40 effectively promoted the expression of γ‐H2AX under X‐ray irradiation, indicating its ability to induce more DSBs to enhance radiotherapeutic efficacy. TUNEL staining revealed a higher level of apoptosis in the inflamed joints treated with the IRnR‐40+RT compared to the monotherapy group, confirming that the combined chemo‐radiotherapy resulted in a more pronounced destruction of pathological synovial tissues. In inflamed synoviums, various inflammatory cytokines are secreted, including tumor necrosis factor‐α (TNF‐α), interleukin‐1β (IL‐1β), interleukin‐6 (IL‐6), which activate osteoclasts and contribute to bone destruction.^[^ ^45^ ^]^ Specifically, the levels of these pro‐inflammatory cytokines were significantly increased in the saline group. However, the group treated with IRnR‐40 and RT showed the lowest levels of these cytokines due to the ROS‐enhancing synergistic chemo‐radiotherapy that efficiently achieves the removal of the hyperplastic synovium and the prevention of secondary inflammatory responses for severe RA. Moreover, the IRnR‐40+RT group demonstrated superior total antioxidant capacity (T‐AOC) due to the synergistic antioxidant activity of IRnR‐40 and RT (Figure 5j). Taken together, these results demonstrated that the synergistic safe‐dose chemo‐radiotherapy could effectively inhibit synovitis in inflamed joints, thereby protecting the articular cartilage and preventing bone erosion. This therapeutic effect could be attributed to the NIR‐II FL imaging‐guided targeted chemo‐radiotherapy for abnormally proliferative synovium.

**Figure 6:**
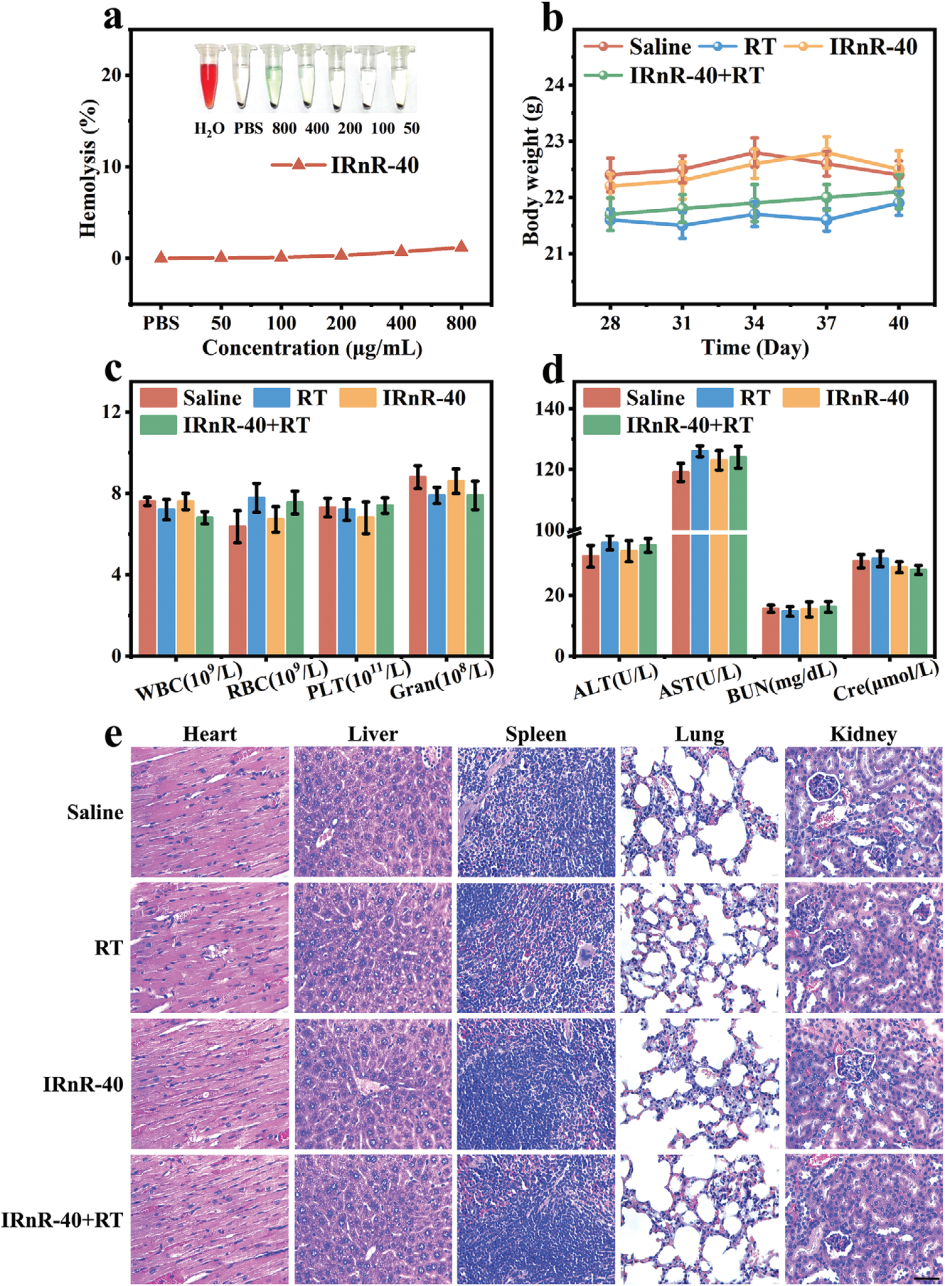
The biosafety of IRnR‐40. a) Hemolysis analysis of IRnR‐40. b) The body weight changes of mice in different groups. c) The blood routine analysis of mice in various groups. d) The blood biochemical examination, including the analysis of alanine aminotransferase (AST), aspartate aminotransferase (ALT), blood urea nitrogen (BUN), and creatinine (Cre) of mice in various groups. e) H&E staining images of the major organs harvested from mice. Scale bar: 50 µm. Mean ± SD (n = 5).

Finally, we comprehensively assessed the biosafety of IRnR‐40, which was essential for biomedical applications. To evaluate the safety of intravenous administration of IRnR‐40, a hemolysis experiment was first conducted. The results demonstrated that the IRnR‐40 did not cause any hemolysis at different concentrations, confirming its good biocompatibility in vitro (**Figure** **6**a). The weight of the mice in different treatment groups remains relatively stable over time as shown in Figure 6b. Moreover, the mice were euthanized for blood routine and blood biochemical analyses 48 h after the IRnR‐40 injection (Figure 6c,d). Compared to the saline group, there were no significant differences observed in blood routine parameters or key liver and kidney function indicators in the IRnR‐40+RT group. The absence of obvious harm to the major organs, like the heart, liver, spleen, lung, and kidney, suggested that the IRnR‐40 had good biosafety in vivo (Figure 6e). Taken together, these results highlight that the MMP‐9‐activatable IRnR‐40 can be safely applied as an excellent nanoradiosensitizer for precisely safe‐dose chemo‐radiotherapy of RA.

## 3 Conclusion

In summary, we have successfully developed a novel enzyme‐activatable nanoradiosensitizer IRnR‐40 to achieve precisely responsive NIR‐II FL imaging‐guided synergistic chemo‐radiosynovectomy for severe RA. Utilizing the clinically approved drugs DDP and ICG, coupled with a biodegradable endogenous substance gel as a nanocarrier, the IRnR‐40 can be mass‐produced with good biosafety for the clinical application of RA. Upon exposure to elevated MMP‐9 concentration in the RA microenvironment, the IRnR‐40 experienced rapid structural decomposition with superior specificity and sensitivity, resulting in the controllable release of functional small molecules (ICG and DDP). The released ICG could specifically illuminate the inflamed joints in RA, enabling accurate delineation of the target volume for guiding radiotherapy and real‐time monitoring of DDP release. We systematically explored the effect of chemotherapy and radiosensitization with DDP on the advanced stage of RA, unraveling the associated mitochondrial caspases‐cascade pathway of ROS‐mediated cell apoptosis to achieve effective synovectomy. The synergistic chemo‐radiotherapy strategy significantly inhibited the progression of RA and yielded no apparent systemic toxicity. Taken together, this activatable IRnR‐40 represents a pioneering step toward achieving efficient NIR‐II FL imaging‐guided safe‐dose chemo‐radiotherapy for severe RA, which also provides a novel paradigm for precise theranostics of refractory benign diseases.

## 4 Experimental Section

Materials and experimental details are provided in the Supporting Information. All animal studies were performed in the Animal Experiment Center of Shanxi Medical University and the procedures involving experimental animals were in accordance with protocols approved by the Institutional Animal Care and Use Committee of the Animal Experiment Center of Shanxi Medical University (No. 2016LL141, Taiyuan, China).

### Statistical Analysis

Statistical analyses were performed using SPSS software (version 25, IBM, Armonk, NY, USA). Data were expressed as mean ± standard deviation (SD). Statistical significance was assessed via the two‐tailed unpaired Student's t‐test. All n‐values per group were reported in the figure legends. No statistical methods were used to predetermine sample size in the study. A *p*‐value of <0.05 was considered to be statistically significant (^*^ *p* < 0.05, ^**^ *p* < 0.01, ^***^ *p* < 0.001), and a *p*‐value of ≥ 0.05 indicated that the differences were not significant (ns).

## Conflict of Interest

The authors declare no conflict of interest.

## Supporting information

Supporting Information

## Data Availability

Research data are not shared.
