# Supplementary material for: A Novel Activatable Nanoradiosensitizer for Second Near‐Infrared Fluorescence Imaging‐Guided Safe‐Dose Synergetic Chemo‐Radiotherapy of Rheumatoid Arthritis
Source: Adv Sci (Weinh). 2024 Feb 28;11(17):2308905. doi: 10.1002/advs.202308905 (PMC11077689; doi:10.1002/advs.202308905)
Supplement: Supplementary file 1 — Supporting Information [file ADVS-11-2308905-s001.pdf]

ADVANCED SCIENCE
Open Access

Supporting Information

for *Adv. Sci.*, DOI 10.1002/advs.202308905

A Novel Activatable Nanoradiosensitizer for Second Near-Infrared Fluorescence Imaging-Guided Safe-Dose Synergetic Chemo-Radiotherapy of Rheumatoid Arthritis

Yarong Jin, Dongsheng Li, Xiaochun Zheng, Mengting Gao, Wenxuan Wang, Xin Zhang, Weiwei Kang, Chongqing Zhang, Shutong Wu, Rong Dai, Ziliang Zheng* and Ruiping Zhang**

Supporting Information

A Novel Activatable Nanoradiosensitizer for Second Near-Infrared Fluorescence Imaging-Guided Safe-Dose Synergetic Chemo-Radiotherapy of Rheumatoid Arthritis

Yarong Jin[#], Dongsheng Li[#], Xiaochun Zheng[#], Mengting Gao[#], Wenxuan Wang, Xin Zhang, Weiwei Kang, Chongqing Zhang, Shutong Wu, Rong Dai, Ziliang Zheng*, and Ruiping Zhang**

1. Experimental Section

1.1. Chemicals

A-gelatin, indocyanine green (ICG), hydrochloric acid (HCl), dimethylformamide (DMF), glutaraldehyde, and matrix metalloproteinase-9 (MMP-9) were purchased from Aladdin Biochemical Technology (Shanghai, China). Cisplatin (DDP) was purchased from Beyotime (Shanghai, China). Chicken type-II collagen, complete Freund's adjuvant, and incomplete Freund's adjuvant were supplied from Chondrex, Inc. (Washington, USA). Deionized water was used in all experiments. All agents were used directly without further purification.

1.2. Characterization

Transmission electron microscope (TEM) and element mapping of samples were carried out on JEM-2100F (Jeol, Japan). Dynamic light scattering (DLS) and zeta potential measurements were conducted using a Zetasizer (Malvern Instruments Ltd., UK). The X-ray photoelectron spectra (XPS) were recorded by a spectrometer (Thermo Scientific K-Alpha, USA) with Al Ka radiation as the excitation source. Fourier-transform infrared (FTIR) spectroscopy was carried out using the KBr pellet technique on a spectrophotometer (Bruker Tensor II, Germany) to identify and quantify the relative abundance of bonds. The TU-1901 dual-beam UV-vis spectrophotometer (PerkinElmer, USA) was used to acquire the UV-vis absorption of the samples. The NIR-II fluorescence emission spectrum was obtained using a fluorescence spectrometer (Suzhou NIR-Optics Co., Ltd., China) with an 808 nm excitation laser source.

1.3. Synthesis of IRnR-40

A-gelatin (11.25 mg) was dissolved in deionized water (5 mL) and stirred at 40 °C for 15 min to obtain a clear solution. DDP (5 mg/mL) dispersed in DMF solution and ICG (1 mg/mL) aqueous solution were added successively to the above solution under constant stirring at 40 °C. The pH value of the solution was adjusted by adding 200 μL of 0.2 mol/L HCl. Then, the resulting products were cross-linked by adding 8.5 μL of 25% glutaraldehyde aqueous solution dropwise and stirring at 40 °C for 6 h in the dark. Finally, the as-synthesized IRnR nanomedicines were collected by ultra-filtered centrifugation and freeze-dried overnight. IRnR with different DDP/ICG mass ratios (DDP/ICG=60/1, 50/1, 40/1, 30/1) were synthesized by adjusting the amount of DDP and ICG.

1.4. *In vitro* test

1.4.1. The stability evaluation of IRnR-40

The size and zeta potential of IRnR-40 were measured in different solutions including deionized water, PBS, Dulbecco's modified Eagle's medium (DMEM), and fetal bovine serum

(FBS), and the particle size and zeta potential changes were measured at 24, 48, 72, and 96 h to assess their stability.

1.4.2. Drug loading and encapsulation efficiency

The drug loading (DL) yields and encapsulation efficiency (EE) of ICG and DDP were determined by measuring their absorbance at 780 nm and 309 nm respectively using a UV-vis spectrophotometer, and comparing the results to standard curves of the free ICG and DDP. The DL and EE were calculated based on Equations (1) and (2), respectively:

DL (%) = Weight of loaded drug/Total weight of IRnR-40 × 100% (1)

EE (%) = Weight of loaded drug/Total weight of feeding drug × 100% (2)

1.4.3. In vitro release of drugs

The release profile of ICG and DDP drugs from the IRnR-40 was determined by dialysis. Specifically, 1 mg of IRnR-40 was resuspended in 1 mL PBS (pH 7.4) with or without MMP-9 (200 μM). The suspension was then placed into a dialysis bag (8000 Da) containing 20 mL of PBS and incubated at 37 °C with constant stirring at 800 rpm. Samples were collected at specific time points (0, 15, 30, 60, 120, and 240 min) from the solution outside the dialysis bag and replaced with fresh PBS buffer of the same pH value. The drug concentrations in the release solution were measured using a UV-vis spectrophotometer, while the Pt concentration was determined using inductively coupled plasma-optical emission spectrometry (ICP-OES, Agilent 5110) analysis.

1.5. Cell lines

1.5.1. Cell culture

Human normal liver cell line (L02) and rheumatoid arthritis synovial fibroblasts (RASF) were acquired from the American Type Culture Collection (Virginia, USA). These cells were cultured in DMEM medium supplemented with 10% FBS and 1x penicillin/streptomycin in a 5% CO_2 incubator at 37 °C.

1.5.2. Gelatin zymography

MMP-9 activity in L02 and RASF cell lines was measured using the gelatin zymography method. First, cells were incubated in a serum-free medium. The medium containing the respective cells was harvested, centrifuged to remove the residues by 2000 rpm for 10 min, and concentrated with Amicon Ultra 30 KD centrifugal filters (Millipore, MA, USA). Then, 20 μL of the clarified supernatant mixed with 5× loading buffer were loaded on an 8% polyacrylamide gel containing 1% gelatin. After electrophoresis, the gel was placed in an enzyme-renaturing solution for 60 min, followed by a reaction in incubation buffer for 42 h at

37 °C. Next, the gel was stained with Coomassie brilliant blue solution for 3 h, resulting in transparent bands as the positive areas on a blue background. To determine the gelatinolytic activity of MMP-9 (~92 kDa), densitometric analysis was performed using Image J software (Bethesda, MD, USA).

1.5.3. Cellular uptake test

L02 and RASF cells were seeded in 48-well plates (1.5×10^4 cells/well) and incubated at 37 °C for 24 h. Then, cells were treated with IRnR-40 and incubated at 37 °C for 1, 2, and 4 h, respectively. Subsequently, cells were washed with PBS buffer and fixed in 4% paraformaldehyde for 30 min. Finally, the cells were imaged using a NIR-II fluorescence microscope.

1.5.4. Cell viability assay

The cytotoxicity of IRnR-40 against L02 and RASF cells was assessed using a standard Cell Counting Kit-8 (CCK-8) (Sigma-Aldrich, St. Louis, USA) assay. The RASF cells were seeded in 96-well plates at a density of 1.0×10^4 cells/well, and then treated with different concentrations of IRnR-40 and a series of radiotherapy (RT) doses. After incubation, the cell's proliferation was immediately measured at a wavelength of 450 nm using a microplate reader (BioTek Epoch 2).

1.5.5. Live/Dead cell staining assay

The RASF cells were divided into four groups and seeded in a 96-well plate. Subsequently, the cells were treated with IRnR-40 (with a concentration of DDP at 4 μg/mL) and RT (1 Gy) for 24 h. Next, the cells were washed with PBS and stained with a dual acridine orange–ethidium bromide (AO/EB) staining kit (Leagene Biotechnology Co., Ltd., China) for 20 min under a fluorescence microscope (Olympus IX73).

1.5.6. Intracellular ROS evaluation

The intracellular ROS levels were detected using the DCFH-DA probe (Solarbio Science & Technology Co., Ltd., China). The RASF cells were seeded in a 96-well plate and incubated at 37 °C overnight. In the material group, the cells were treated with IRnR-40 for 24 h. Then cells were washed with PBS and labeled with the DCFH-DA probe (10 μM in FBS-free DMEM) for 30 min. Subsequently, the RT group was subjected to irradiation (1 Gy). The fluorescence microscope was used to observe the level of intracellular ROS.

1.5.7. Mitochondrial membrane potential (MMP) assay

The change in MMP was measured using the JC-1 kit (Beyotime, China). The RASF cells were seeded in a 96-well plate and treated with IRnR-40 and RT for 24 h. Finally, the JC-1

probe was used to stain the mitochondria, and images were captured under a fluorescence microscope.

1.5.8. Immunofluorescence analysis

Immunofluorescence analysis was utilized to evaluate the levels of DNA damage. RASF cells were seeded onto coverslips and incubated overnight at 37 °C. Once attached, the cells were exposed to IRnR-40 and RT. The cells were then fixed with a 4% polyformaldehyde solution, permeabilized with 0.5% Triton X-100 for 20 min, and blocked with a PBS solution. Subsequently, the cells were incubated with an antibody against γ-H2AX (Ser139) (AF5836; Beyotime) at 4 °C overnight, followed by Alexa Fluor^{TM}594 goat anti-rabbit (R37117; Invitrogen) at 37 °C for 1 h, respectively. DAPI solution (C1005; Beyotime) was used to stain the cell nuclei. Images were captured using a fluorescence microscope.

1.5.9. Protein extraction and western blot

RASF cells were treated with PBS, IRnR-40, RT (1 Gy), and IRnR-40 plus RT for 24 h. Afterward, the cells were collected, lysed with a lysis buffer, and the protein concentrations were determined using a BCA assay kit (Beyotime, China). The proteins were denatured and subjected to SDS-PAGE gel. Antibodies against γ-H2AX (Ser139) (AF5836; Beyotime), Bcl-2 (T40056F; Abmart), Bcl-X_L (T40057F; Abmart), Cytochrome C (T55734S; Abmart), Caspase-9 (T40046F; Abmart), Caspase-3 (T40044F; Abmart), PARP (T40050F; Abmart), GAPDH (60004-1-lg; Proteintech) were used overnight at 4 °C. Appropriate secondary antibodies (Beyotime, China) were used at room temperature for 2 h. The protein bands were visualized by the Odyssey Clx system.

1.5.10. Cell apoptosis assay

RASF cells were treated with PBS, IRnR-40, RT (1 Gy), and IRnR-40 plus RT for 24 h, and subsequently stained using an Annexin V-FITC/PI apoptosis detection kit (Yeasen Biotechnology Co., Ltd., China) following the manufacturer's instructions. Finally, the rate of apoptosis was assessed through flow cytometry (BD, USA).

1.6. Animal

1.6.1. Animal model

DBA/1 female mice aged 6-8 weeks were purchased from Beijing Vital River Laboratory Animal Technology Co., Ltd. (Beijing, China). A double immunization strategy was applied to establish the collagen-induced arthritis (CIA) model. For the first immunization, the mice were injected intradermally on their back with an emulsion containing an equal volume of chicken type-II collagen solution (2 mg/mL) and complete Freund's adjuvant (4 mg/mL). After 21 days of the first immunization, the mice received a boost immunization, in which

they were injected with an emulsion of chicken type-II collagen solution and incomplete Freund's adjuvant. The success of the model was evaluated by assessing the inflammatory scores of the mice's joints. All animal procedures followed the criteria of the National Regulation of China for Care and Use of Laboratory Animals and were approved by the Institutional Animal Use and Care Committee of Shanxi Medical University (Approval No, 2016LL141, Taiyuan, China).

1.6.2. In vivo and ex vivo fluorescence imaging

To visualize the biodistribution of IRnR-40 *in vivo*, fluorescence imaging experiments were conducted. The CIA mice were anesthetized, intravenously administered with IRnR-40 (5 mg/kg), and then imaged at different time points (0, 2, 4, 6, 12, and 24 h). The healthy mice conducted the NIR-II FL imaging were regarded as the control group. The mice from healthy and RA groups were sacrificed 6 h after injection. We then collected, imaged, and analyzed the *ex vivo* major organs (hearts, livers, spleens, lungs, and kidneys) and joints of mice. The NIR-II FL imaging was performed using a series II900/1700 *in vivo* imaging system (Suzhou NIR-Optics Co., Ltd., China).

1.6.3. Micro-CT measurement

Micro-CT (SkyScan 1276, Bruker, German) was employed to evaluate the bone erosion or destruction of the inflamed joints of CIA mice. Next, 3D tomograms were reconstructed and quantitative analyses of the interest region were conducted using the software CTAN.

1.6.4. In vivo biodistribution assay

To determine the distribution of IRnR-40 *in vivo*, we obtained hearts, livers, spleens, lungs, kidneys, and inflamed joints 6 h after administering the IRnR-40. The tissue samples were then digested with HNO_3 at 80 °C and the amount of Pt element in the solution was analyzed using ICP-OES.

1.6.5. Therapeutic efficacy evaluations in vivo

To study the therapeutic effects of IRnR-40 *in vivo*, we randomly divided CIA mice into four groups (n=5). Six hours after injecting IRnR-40 (5 mg/kg) or saline, the mice were anesthetized and their inflamed joints were irradiated with the X-ray irradiator at 1 Gy per fraction every 3 days starting from day 28 since the first immunization for a total of five times. The clinical scores of the mice were calculated at the end of the treatment. The arthritis scores of four paws were evaluated from 0 to 4 as follows: 0 = absence of erythema or swelling, 1 = erythema and mild swelling, 2 = erythema and mild swelling extending from the ankle to the tarsals, 3 = erythema and moderate swelling extending from the ankle to metatarsal joints, and 4 = erythema and severe swelling encompassing the ankle, foot, and digits or ankylosis of the

limb. The arthritic scores of four paws were summed. The paw thickness was measured using a caliper. The timeline for the animal experiments can be seen in Figure 5a.

1.6.6. Gait analysis

To assess the limb behavior of the affected limb, the mice with red-inked fore paws and blue-inked hind paws were allowed to freely walk from one side to the other without any additional stimulation. The footprints of each mouse were recorded, and evaluated by three independent observers who were blinded to the experiment groups.

1.6.7. Histopathology analysis

To examine the pathological changes of the CIA mice, the joints were fixed in 4% paraformaldehyde for 1 day and then decalcified for 10 days using 10% EDTA. The joints were then embedded in paraffin, sectioned, and stained with hematoxylin and eosin (H&E), and Safranin-O for histological analysis. A microscope (C2 Plus, Nikon, Tokyo, Japan) was used to observe the tissue sections and analyze the histological synovitis score (HSS)[1] to assess the status of synovitis, as well as the modified Osteoarthritis Research Society International (OARSI) score[2] to evaluate the articular cartilage. Immunofluorescence staining was also conducted to measure the expression levels of γ-H2AX, and TUNEL staining was used to assess apoptosis in the joint tissues.

1.6.8. Enzyme-linked immunosorbent assay (ELISA)

The serum obtained from CIA mice was used to detect the levels of pro-inflammatory cytokines, including tumor necrosis factor-α (TNF-α), interleukin-1β (IL-1β), interleukin-6 (IL-6) by using ELISA kits (Shanghai Jianglai Biotechnology Co., Ltd., China).

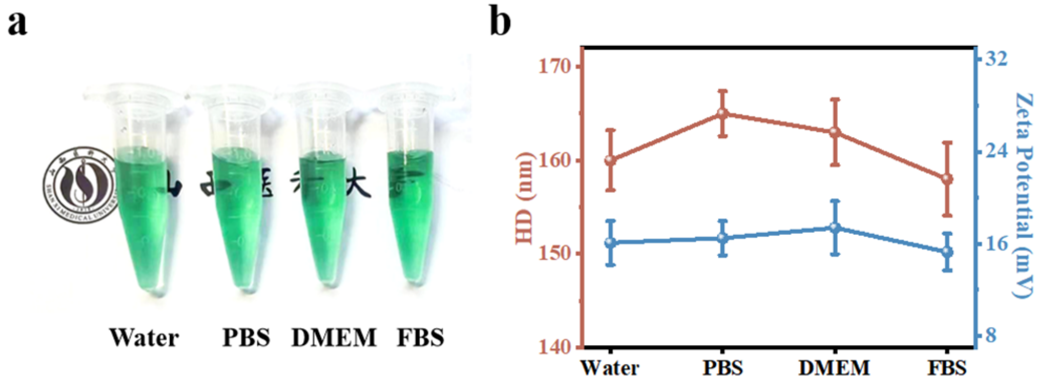

Figure S1. a) Digital photos, b) Hydrodynamic diameter (HD) and zeta potentials of IRnR-40 in various solutions after 24 h.

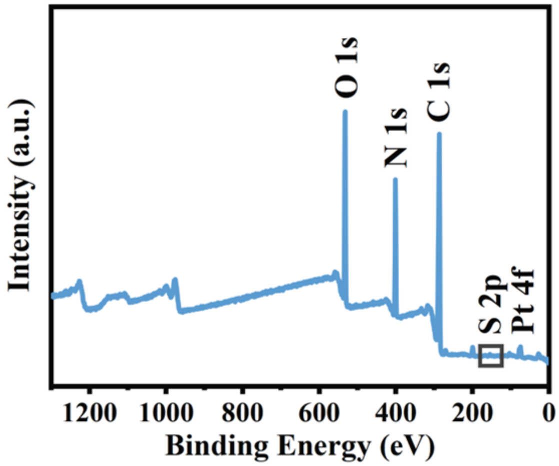

Figure S2. XPS spectrum of IRnR-40.

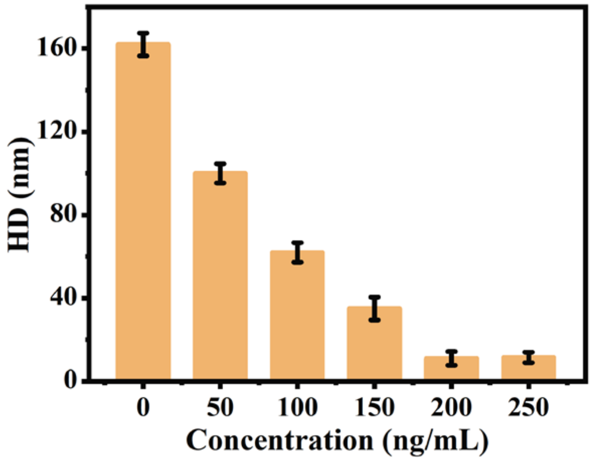

Figure S3. HD changes of IRnR-40 treated with MMP-9 at different concentrations.

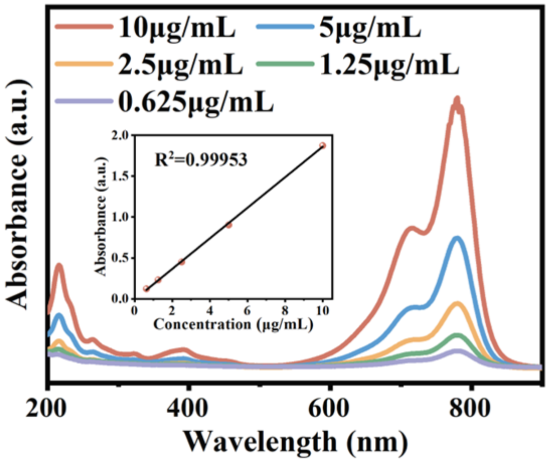

Figure S4. UV-vis absorption of ICG at different concentrations. Inset: the standard curve of the concentration of ICG versus its absorbance.

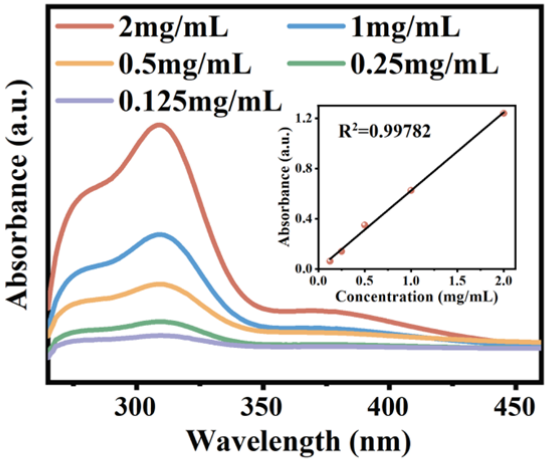

Figure S5. UV-vis absorption of DDP at different concentrations. Inset: the standard curve of the concentration of DDP versus its absorbance.

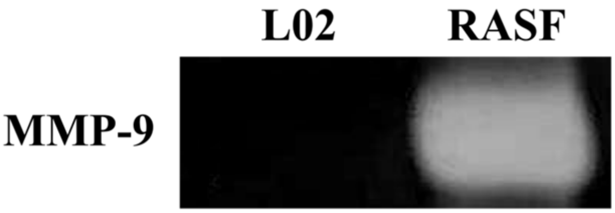

Figure S6. Gelatin zymography analysis of the relative MMP-9 enzyme activities in L02 and RASF cell lines.

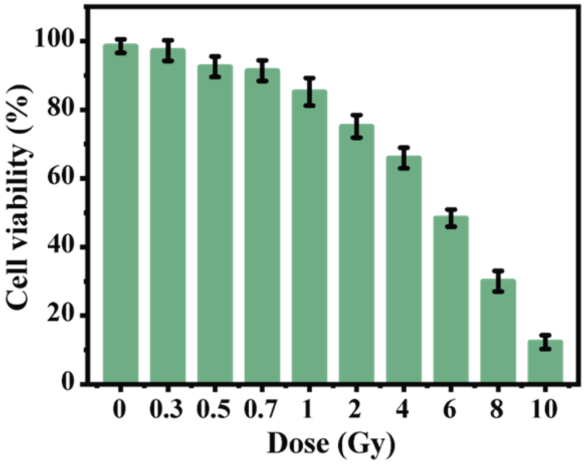

Figure S7. Cell viability of RASF cells subjected to different radiation doses.

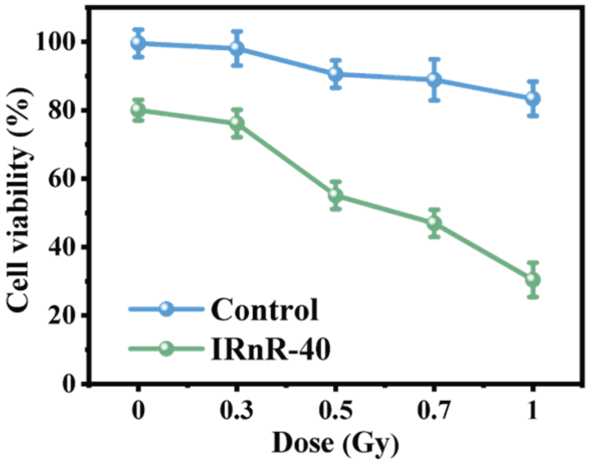

Figure S8. Cell viability of RASF cells after co-incubation with PBS and IRnR-40 at different doses for 24 h.

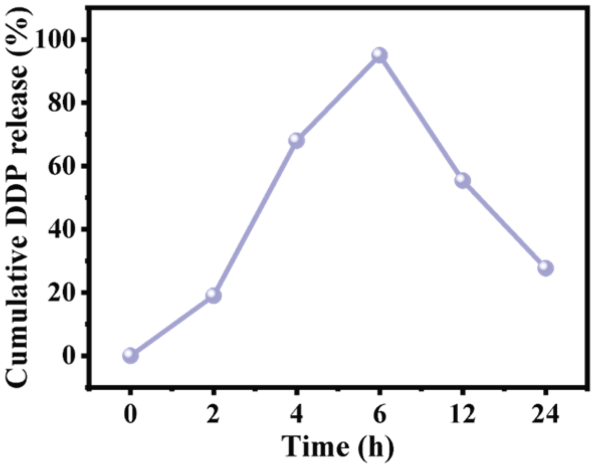

Figure S9. The kinetic curve of DDP released from IRnR-40 injected intravenously in RA mice. The profile was calculated from the linear relationship (y = 0.203x+9.875) between the cumulatively released DDP (x) and the fluorescence intensity of IRnR-40 in the presence of MMP-9 (y).

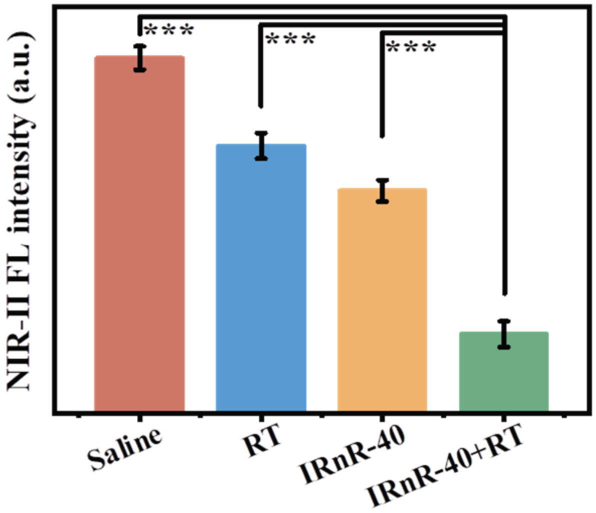

Figure S10. Quantitative analyses of relative NIR-II FL signal in Figure 5b.

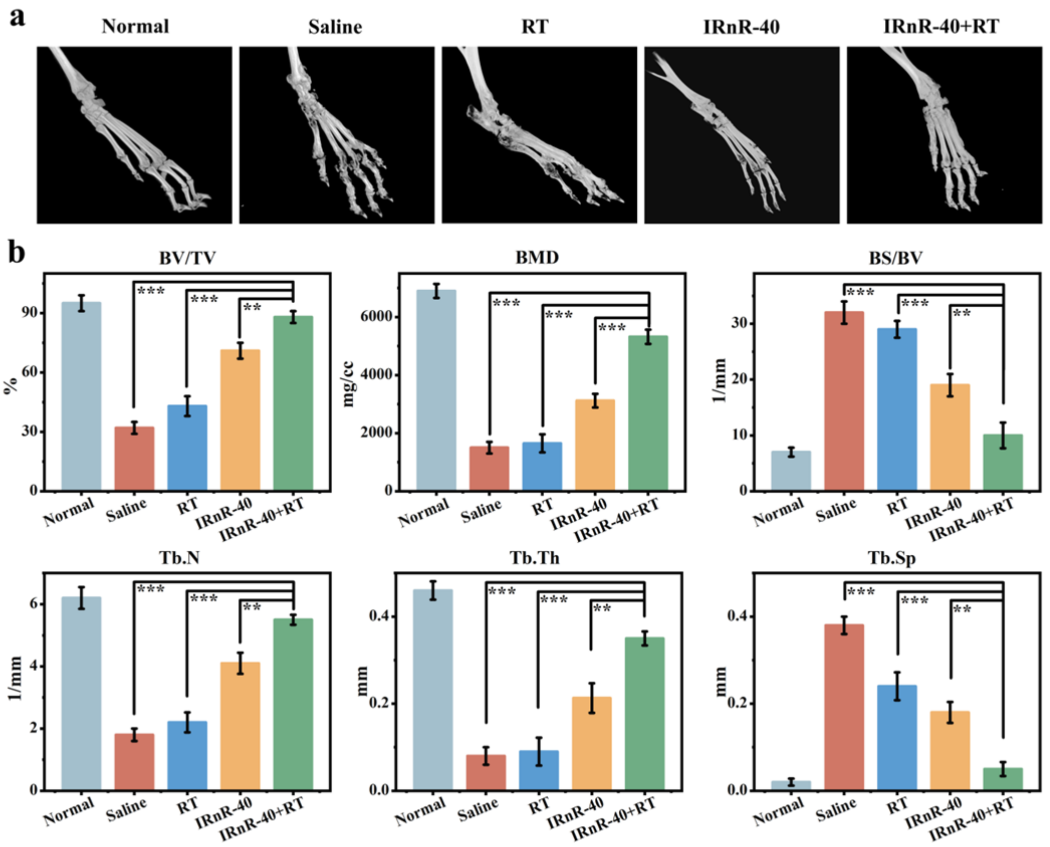

Figure S11. a) Representative micro-CT reconstructed images of the inflamed joints in different groups, b) Quantitative analysis of morphometric parameters of the bone interest region: ratio of bone volume to tissue volume (BV/TV), bone mineral density (BMD), ratio of bone surface area to bone volume (BS/BV), trabecular number (Tb.N), trabecular thickness (Tb.Th), and trabecular separation (Tb.Sp). Mean±SD (n = 5). **P<0.01, ***P<0.001.

References

[1] V. Krenn, L. Morawietz, T. Häupl, J. Neidel, I. Petersen, A. König, *Pathol. Res. Pract.* **2002**, *198*, 317.

[2] K. P. H. Pritzker, S. Gay, S. A. Jimenez, K. Ostergaard, J.-P. Pelletier, P. A. Revell, D. Salter, W. B. van den Berg, *Osteoarthritis Cartilage* **2006**, *14*, 13.
